# Historical and geographical parallelism between the incidence of dental caries, *Streptococcus mutans* and sugar intake

**DOI:** 10.1007/s10654-013-9826-7

**Published:** 2013-07-24

**Authors:** Didier Raoult, Bruno Foti, Gérard Aboudharam

**Affiliations:** 1 Unité de Recherche sur les Maladies Infectieuses et Tropicales Emergentes, Faculté de Médecine, CNRS UMR 7278, IRD 198, Aix-Marseille Université, 13385 Marseille Cedex 5, France; 2 King Abdulaziz University of Science and Technology, Jidda, Saudi Arabia; 3 Unité Mixte de Recherche 7268 A.D.E.S./CNRS-EFS-Aix Marseille Université, 51 Boulevard Pierre Dramard, 13916 Marseille Cedex 20, France

**Keywords:** Dental caries, *Streptococcus mutans*, Paleomicrobiology, Sugar, Anthropology

Currently, the distribution of dental caries in patients over 35 years can be super-imposed to that of the countries where the food industry has developed, with, in particular the addition of sugar in the food diet. Thus, countries with a very high incidence are Western Europe, North America, Australia, Brazil, Chile and Peru. However, in Africa, South and East Asia the incidence is much lower [1]. This will tend to also self correct as preventive measures against dental caries have been taken in children.

Interestingly, one can note that dental caries are associated with a particular oral microbiota associated with said cariogenic bacteria, including *Streptococcus mutans*. We have been working for many years on paleomicrobiology which is the study of the diseases of the past by identifying antigens or DNA trapped bacteria within the dental pulp [2]. A recent work of paleo-microbiology from another team has demonstrated the increased presence of cariogenic bacteria over time [3]. Indeed, the work has focused on the systematic analysis of tartar and dental plaque in skeletons dating from the Mesolithic in our time through the Neolithic, Bronze Age and the Middle-Ages. By studying the PCR amplified sequences of 16S rDNA, a microbiote complex preserved until the nineteenth century was identified. From the nineteenth century, a very sharp decline in the biodiversity of dental bacteria has been observed as well as the onset of the oral microbiota associated with the presence of caries with a predominance of *S. mutans*. This is contemporary to the industrial age and to the sharp increase in the consumption of sugar and sugary drinks, of industrialized countries.

We have reanalyzed the teeth that we have received for paleomicrobiological analysis, especially that of the eighteenth and early nineteenth century. When comparing the oral health status of these skeletons we will find that during the pre-industrial period teeth were in a better condition as out of 110 adults died of the plague in Martigues near Marseille, in a small town in 1720 during the great Plague of Marseille, 12.6 % presented a carious lesion. Out of 113 adults died of the plague in Marseilles in 1,722, 11.69 % of the teeth had carious lesions. Finally, among 146 adults, male only, soldiers of the Grand Army and died in Vilnius in 1,812 only, 8.1 % had at least one carious lesion. In these three cases, the prevalence of caries was lower than it is now recognized in France in patients over 35 years. Another study showed that England was the first country to industrialize the production of sugar, was the first country to experience an increase in the proportion of dental caries [4]. Thus, in the early nineteenth century the English had more dental caries than the American.

Under such conditions, there is an obvious historical link between the dramatic increase in the prevalence of dental caries, and that of *S. mutans*. The latter appears as the consequence of food globalization and industrialization which translated into an increase in the consumption of added sugar in foods. This resulted in an increase in caries in the nineteenth century in industrialized countries. In the twentieth and twenty first century, the number of caries is increasing in the countries and regions affected most recently by globalization [5]. This should be an element adding to the debate on the prevention of excessive sugar intake in children to prevent both obesity and dental caries.

Finally, anthropological studies as well as paleomicrobiological works appear to contribute to explaining the current public health problems by analyzing changes in our microbiota, alimentation, bone and teeth lesions.
